# Response surface optimization of culture medium for enhanced docosahexaenoic acid production by a Malaysian thraustochytrid

**DOI:** 10.1038/srep08611

**Published:** 2015-02-27

**Authors:** Vidyah Manikan, Mohd Sahaid Kalil, Aidil Abdul Hamid

**Affiliations:** 1 School of Biosciences and Biotechnology, Faculty of Science and Technology, Universiti Kebangsaan Malaysia, 43600 Bangi, Malaysia; 2 Department of Chemical and Process Engineering, Faculty of Engineering and Built Environment, Universiti Kebangsaan Malaysia, 43600 Bangi, Malaysia

## Abstract

Docosahexaenoic acid (DHA, C22:6*n-3*) plays a vital role in the enhancement of human health, particularly for cognitive, neurological, and visual functions. Marine microalgae, such as members of the genus *Aurantiochytrium*, are rich in DHA and represent a promising source of omega-3 fatty acids. In this study, levels of glucose, yeast extract, sodium glutamate and sea salt were optimized for enhanced lipid and DHA production by a Malaysian isolate of thraustochytrid, *Aurantiochytrium* sp. SW1, using response surface methodology (RSM). The optimized medium contained 60 g/L glucose, 2 g/L yeast extract, 24 g/L sodium glutamate and 6 g/L sea salt. This combination produced 17.8 g/L biomass containing 53.9% lipid (9.6 g/L) which contained 44.07% DHA (4.23 g/L). The optimized medium was used in a scale-up run, where a 5 L bench-top bioreactor was employed to verify the applicability of the medium at larger scale. This produced 24.46 g/L biomass containing 38.43% lipid (9.4 g/L), of which 47.87% was DHA (4.5 g/L). The total amount of DHA produced was 25% higher than that produced in the original medium prior to optimization. This result suggests that *Aurantiochytrium* sp. SW1 could be developed for industrial application as a commercial DHA-producing microorganism.

Docosahexaenoic acid (DHA, 22:6*n-3*) is an essential polyunsaturated fatty acid (PUFA) required by most vertebrates. In humans, this PUFA has been implicated in the prevention of several diseases, including cardiovascular^1^ and neurological^2^ diseases. Current commercial sources of DHA are fish and fish oil. However, this fatty acid, partially due to dietary preferences, is conveniently ingested through supplements or enriched foods. These methods of administration are especially preferred by populations whose diets are low in seafood^3^. According to a report by Ward and Singh^4^, a daily dietary intake of 1 g DHA derived from seafood would require the consumption of a large quantity of fish. Moreover, there are possible health risks, such as food poisoning and allergies, associated with the consumption of seafood and fish oils, particularly when there are issues of seawater contamination or toxicity and outbreaks of fish diseases. The risks also include contamination of the fish supply in particular areas and the presence of allergens, which may cause adverse effects to human health, in certain species. These factors often drive consumers' choices away from seafood and related products^3^. Indeed, health advisories often warn consumers about the risks of consuming fatty fish and large predatory fish, which are particularly prone to contamination. Environmental pollutants, which are hydrophobic in nature, accumulate along the marine food chain, particularly in the lipid depots of fish^5^. In addition, global catches have been declining steeply over the last three decades, and the number of overfished stocks has been increasing exponentially since the 1950s^6^,^7^. The expanding requirements for DHA worldwide are now placing pressure on both fisheries and the fish oil supply^8^. Therefore, efforts have been made to find alternative sources for omega-3 fatty acid production, such as thraustochytrids and marine diatoms^9^.

Thraustochytrids are cosmopolitan apochlorotic stramenopile protists that are classified in the class *Labyrinthulomycetes* within the kingdom *Chromista*^10^,^11^,^12^. This group of organisms is unique in that they produce high biomass in culture, with a high proportion of lipids, including a high proportion of PUFAs, particularly of the omega-3 series such as DHA^13^. They are a crucial food resource for higher organisms in marine systems^15^,^16^,^17^, owing to their high productivity in manufacturing DHA^14^. For this reason, thraustochytrids are receiving much attention as a potential source of enrichment for food and feed^18^ and as a prospective alternative to fish oil as the main source of DHA. It has long been known that environmental factors are important determinants of the quantity and quality of lipids produced by microalgae such as thraustochytrids^19^. As such, medium composition may have a decisive role in determining the quantity and quality of DHA produced by thraustochytrids^20^. The production and storage of lipids by microalgae in response to environmental factors are species-specific^19^.

In our previous study, a recently isolated Malaysian strain of thraustochytrid, *Aurantiochytrium* sp. SW1 (previously known as *Schizochytrium* sp. SW1), was found to contain high amounts of lipid with a PUFA content of more than 50%, of which more than 90% was DHA, under non-optimal conditions. Using factorial screening experiments, glucose, yeast extract, monosodium glutamate (MSG) and sea salt were identified as significant medium components that play vital roles in promoting high biomass production as well as high lipid and DHA accumulation by this strain^21^. Following the screening experiment, this study was carried out to optimize the levels of these components and investigate their effects on lipid and DHA production using response surface medium optimization.

## Results

### Effects of medium components on lipid

The amount of lipid accumulated by SW1 varied widely, ranging from 0.24 to 10.41 g/L culture medium, with a maximum to minimum ratio of 44.09. A ratio greater than 10 usually implies that a power transformation is needed to increase the normality of the dataset. However, the normal plot of residuals confirmed that the dataset follows a normal distribution and therefore analysis of variance (ANOVA) was carried out without any power transformation. Based on the sequential model sum of squares, a quadratic equation was chosen as the fitted model, being the highest order unaliased polynomial with the lowest probability (P) value, insignificant lack of fit and highest R^2^.

The F- and P-values were used to identify the effect of each factor on the amount of lipid accumulated. From the partial sum of square analysis (as shown in Table 1), it was found that glucose, yeast extract and sea salt as well as the interaction between yeast extract and sea salt are significant model terms in determining the amount of lipid produced by SW1. The F- and P-values of each factor show that yeast extract has the largest effect on lipid production, followed by sea salt, the interaction between yeast extract and sea salt, and glucose.

The experimental values obtained from the central composite design (CCD) were regressed using a quadratic polynomial equation, and the two regression equations, expressed in terms of the coded and actual factors, are shown below.

Final equation in terms of coded factors:
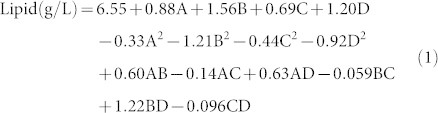


Final equation in terms of actual factors:
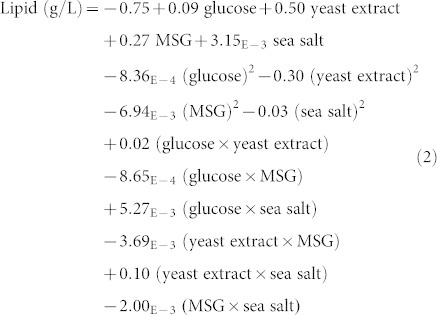


### Effects of medium components on DHA

The highest percentage of DHA was produced in standard run number 8, where DHA accounts for 48.6% of the total lipid, whereas the lowest percentage was produced in standard run number 29 (14.5% of total lipid). The ratio of maximum to minimum is 3.34, showing that a power transformation step is not necessary. In accordance with this ratio, the normal plot of residuals showed that the dataset follows a normal distribution. Similar to the response of lipid discussed above, the sequential model sum of squares, lack of fit test and R^2^ value showed that a quadratic equation is the suitable model for regression of the experimental data. Upon choosing the fitted model, an ANOVA was conducted to obtain the F and P-values for the factors involved.

Table 2 shows the ANOVA results for DHA accumulation. From this table, it can be observed that MSG and sea salt as well as the interaction between yeast extract and sea salt have significant effects on the percentage of DHA in lipid accumulated by SW1. The order of effects of significant factors on percentage of DHA is: sea salt > interaction between yeast extract and sea salt > MSG. The final equations for the response of DHA in terms of the factors are given below:

Final equation in terms of coded factors:
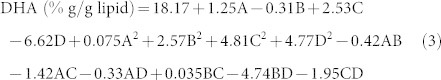
Final equation in terms of actual factors:
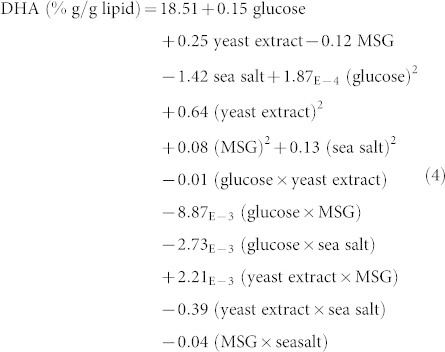


### Medium optimization for enhanced lipid and DHA production

Software-based numerical optimization of the overall desirability function was performed to simultaneously determine the best possible goals for each response. The predicted optimal values for the variables were as follows: A = 60.00 g/L, B = 2.00 g/L, C = 24.00 g/L and D = 6.00 g/L. This combination was predicted to yield 9.49 g/L lipid with 41.06% DHA content. To examine the validity of this prediction, three successive experiments were performed using the predicted optimal medium. The average amount of lipid obtained in these experimental runs was 9.55 g/L with a DHA content of 44.07%. Overall, the absolute amount of DHA produced was 4.21 g/L, near to the predicted result of 3.90 g/L.

### Scaling-up of DHA production

**Figure 1:**
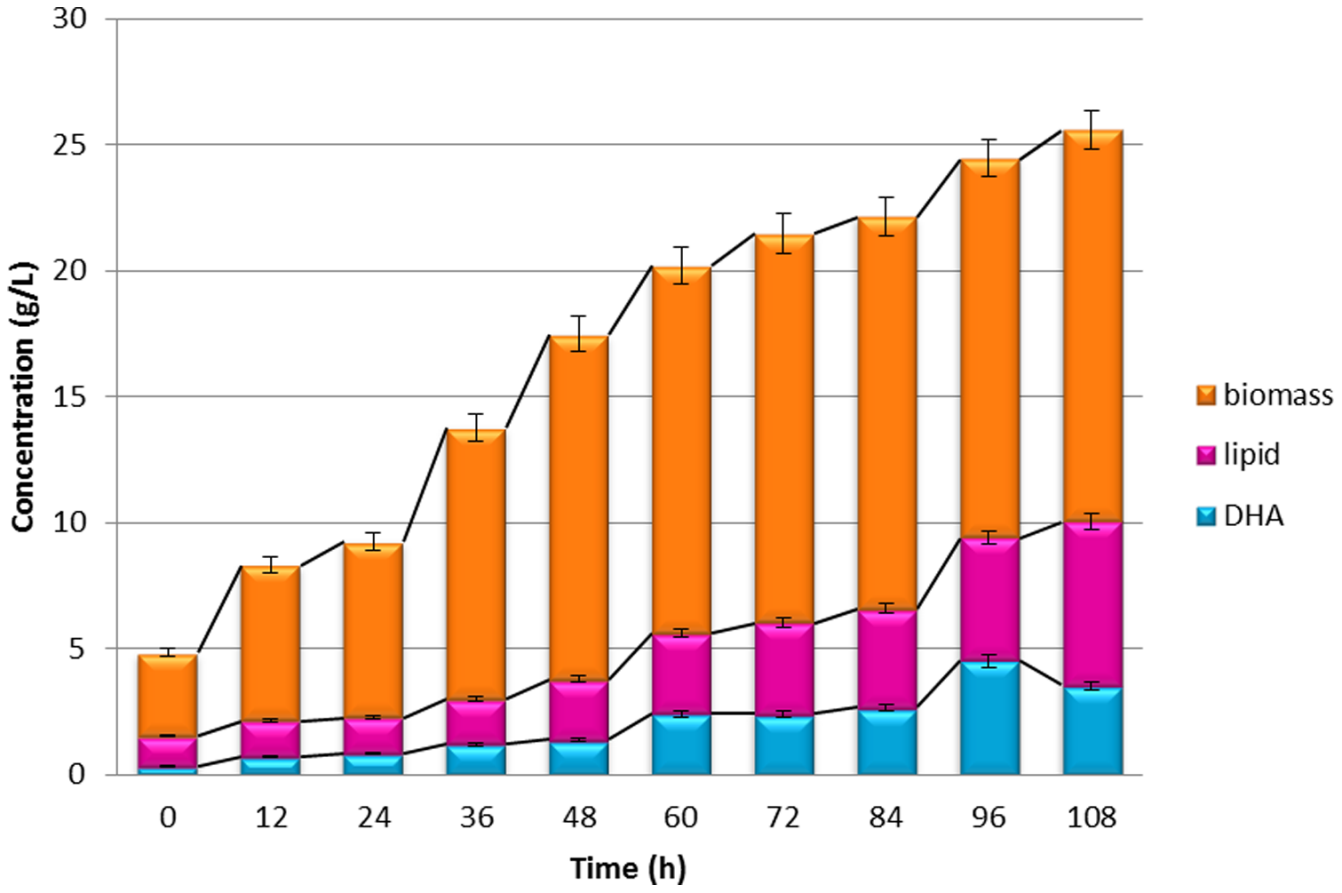
Growth, lipid and DHA profiles of *Aurantiochytrium* sp. SW1 (grown in 5 L bench-top bioreactor at 30°C, agitation rate of 200 rpm and aeration rate of 1 vvm).

The optimized medium was used in a 5 L bench-top fermenter with a working volume of 3 L to assess its applicability and reproducibility on a larger scale. In this run, 9.40 g/L lipid was produced with a DHA content of 47.92%. Figure 1 shows the growth, lipid and DHA profiles of SW1 in this bioreactor run. The absolute amount of DHA accumulated was 4.50 g/L, close to that obtained in shake flask runs. This value is 25% higher than that produced in the original non-optimized medium.

## Discussion

**Figure 2:**
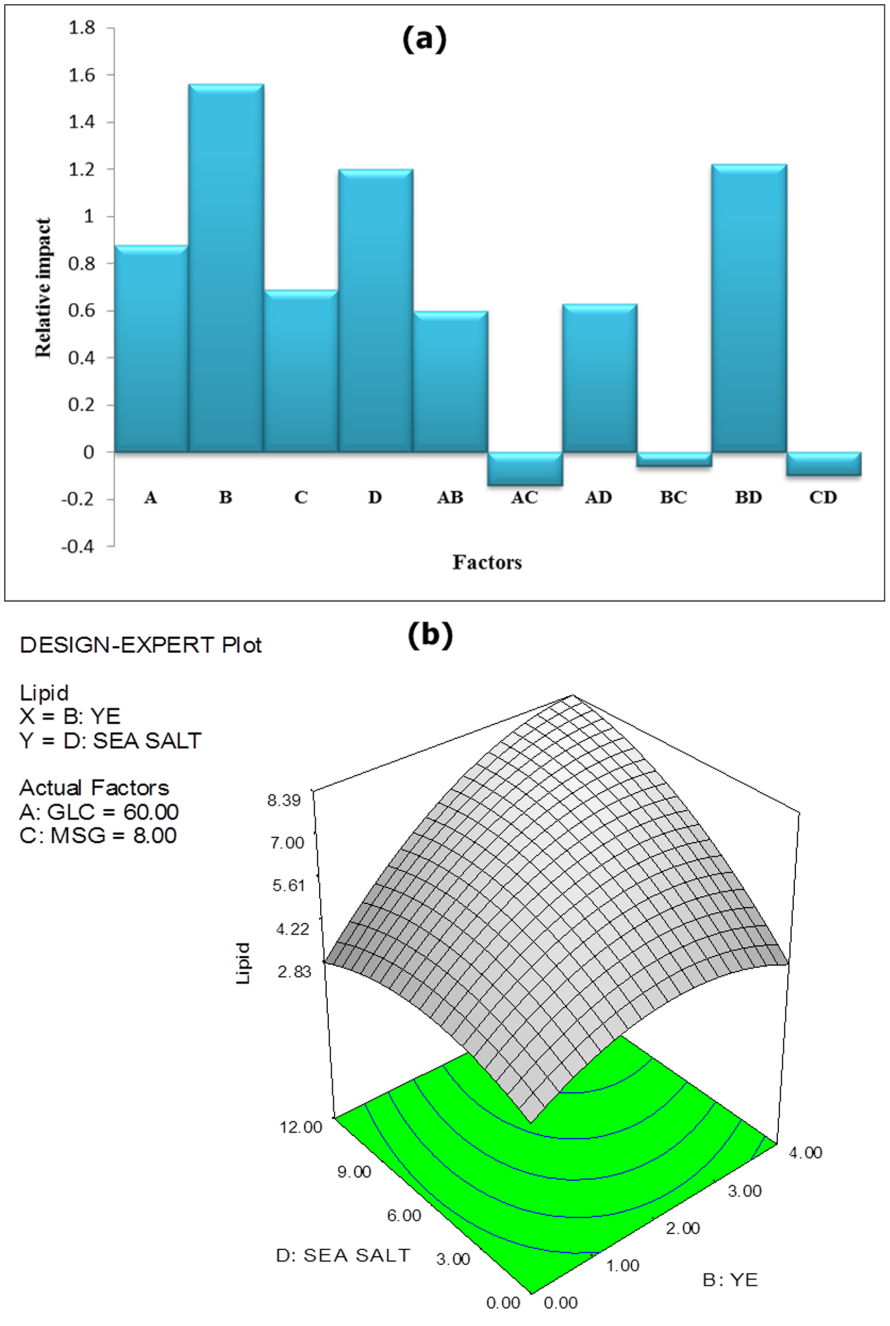
Graphs illustrating (a) relative effect of factors on lipid accumulation, and (b) effect of yeast extract-sea salt interaction on lipid accumulation.

According to Anderson and Whitcomb^22^, the coefficients obtained in the final equation (in terms of coded factors) can be directly compared to assess the relative impact of factors. Figure 2a illustrates the relative effects of factors affecting the amount of lipid accumulated by SW1 via a bar graph. It can be concluded that the four significant factors, namely glucose (A), yeast extract (B), sea salt (D) and the interaction between yeast extract and sea salt (BD) have profound effects on lipid accumulation compared to other factors studied.

It is well known that the amounts of carbon and nitrogen supply have profound effect on the amount of biomass produced. Apart from the carbon and nitrogen supply, the availability of trace minerals, particularly iron (Fe) and zinc (Zn), also affect the biomass production of thraustochytrids to a considerable extent^23^. In this study, although trace minerals are not supplied as separate components, sea salt serves as a source of the essential minerals required for optimal growth of SW1. The results of inductively coupled plasma mass spectrometry (ICP-MS) showed that sea salt solution contains a vast variety of minerals. Table 3 lists the minerals found at concentrations above 0.05 mg/L in full strength sea salt solution (35 g sea salt/L).

Optimal cell growth consequently leads to high absolute amount of lipid (g/L) although in some cases, the relative amount of lipid accumulated per unit biomass (% g/g biomass) is not high. Thus, it is reasonable that the three aforementioned factors which best favor growth are statistically significant in affecting the absolute amount of lipid produced by SW1. Conversely, the highly significant interaction between yeast extract and sea salt might imply that SW1 has an absolute requirement for vitamins provided by yeast extract and minerals provided by sea salt to accumulate high amounts of lipid. The pattern of the effect of this interaction on lipid can be visualized using a three-dimensional response surface plot and corresponding contour plot, as shown in Figure 2b.

This type of graphical visualization allows the relationships between the experimental levels of each factor and the response to be investigated, and the type of interactions between test variables to be determined, which is necessary to establish the optimal medium components and culture conditions. The elliptical nature of the curves shown in Figure 2b (as opposed to circular shapes) indicates significant mutual interactions between the variables (yeast extract and sea salt)^24^. At low to moderate levels of yeast extract concentration, the lipid concentration is unaffected by increasing levels of sea salt. However, at high yeast extract concentrations, an increase in sea salt concentration leads to a significant increase in the amount of lipid. This indicates that the effect of sea salt on lipid is dependent on the levels of yeast extract provided.

**Figure 3:**
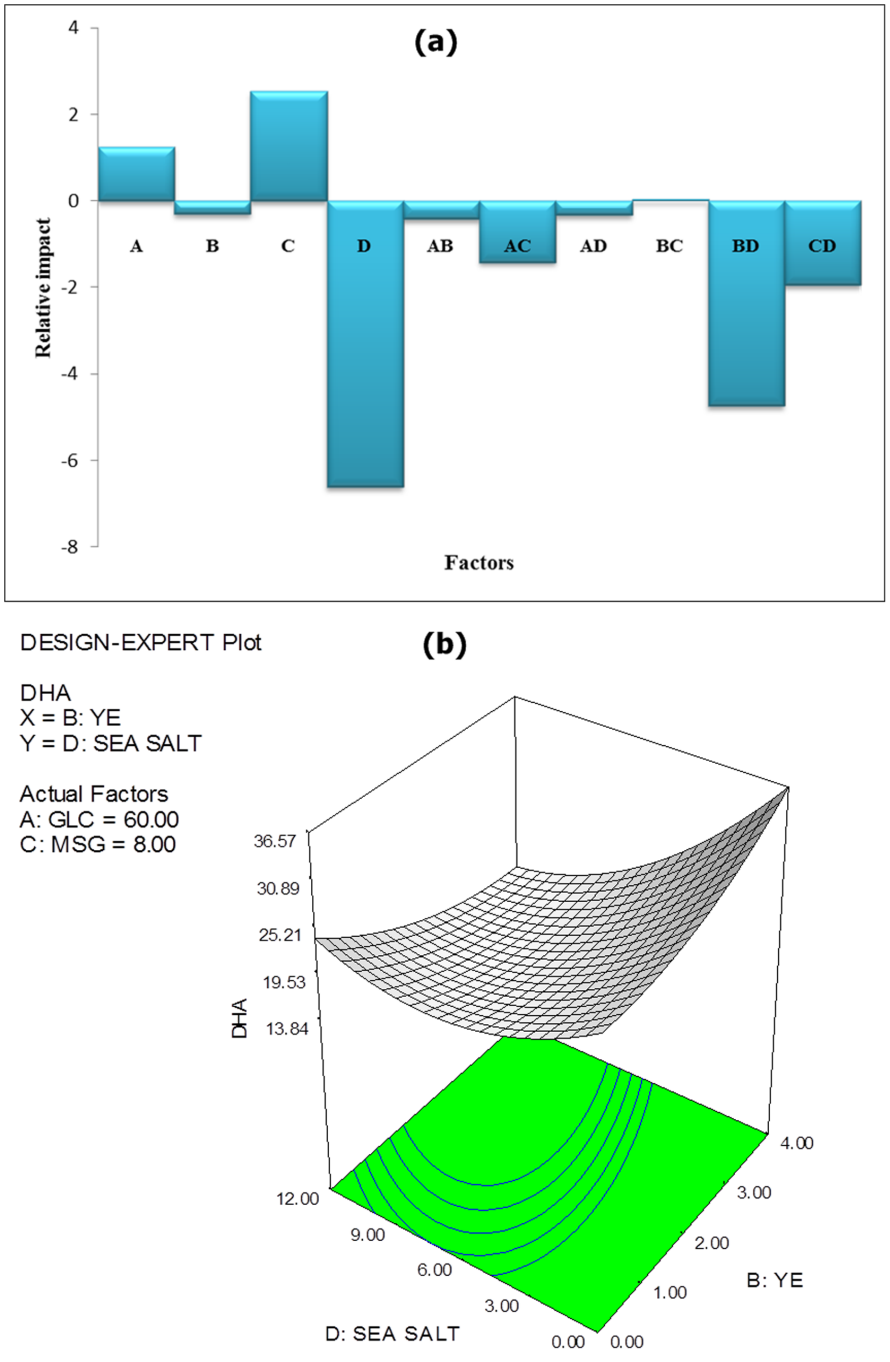
Graphs illustrating (a) relative effect of factors on DHA content, and (b) effect of yeast extract-sea salt interaction on DHA content.

It is noteworthy that yeast extract, which has greatest influence on lipid accumulation, is not a significant factor for DHA accumulation. Notably, MSG, which had no significant effect on lipid, exerts considerable effect on DHA concentration. Similar results were reported by Burja et al.^25^, where they found that the addition of MSG to culture medium resulted in enhanced DHA production by *Thraustochytrium* sp. ONC-T18. In agreement with this study, Ren et al.^26^ reported that MSG positively influenced DHA accumulation in *Schizochytrium* sp. CCTCC M209059. Interestingly, in contrast to lipids, most factors exert negative effects on the percentage of DHA, whereas only glucose and MSG have positive effects (as shown in Figure 3a). This signifies that percentage of DHA accumulation is a subtle response, which requires very detailed and careful analyses to be optimized successfully.

Similar to lipids, the percentage of DHA was significantly affected by the interaction of yeast extract with sea salt (BD). However, in contrast to its effect on lipids, this interaction affects DHA negatively, as indicated by a negative coefficient for this factor in the coded term equation. Figure 3b shows the three-dimensional response surface plot for this interaction. From this figure, it can be deduced that providing high levels of both yeast extract and sea salt simultaneously has a strong negative effect on DHA. Similarly, low levels of both components do not promote DHA accumulation. Thus, to obtain high amounts of DHA, either one of these factors must be kept at a low level while maintaining the other factor at high level.

To date, numerous studies have been conducted to enhance DHA production of thraustochytrids using various modes of fermentation and strategic techniques. Table 4 summarizes biomass production and DHA content of various thraustochytrid strains cultivated using glucose as major carbon source in bench-top bioreactors in comparison to SW1. Among the reports listed in this table, *Aurantiochytrium* sp. KRS101 is one of the strains that have been found to have a high genetic similarity (BLAST) and close phylogenetic relationship with SW1. In an attempt to enhance DHA production by this isolate, Hong et al.^27^ carried out the conventional one-factor-at-a-time (OFAT) optimization where final DHA concentration was successfully increased to 2.8 g/L from the initial concentration of ±2.0 g/L (5 L scale, 72 h). This is equivalent to a DHA production rate of 38.9 mg/L/h. In comparison, SW1 produced 4.5 g/L (5 L scale, 96 h), equivalent to 46.9 mg/L/h in the optimized medium formulated using the more sophisticated statistical design of experiments (DoE).

In conclusion, the production medium for enhanced DHA accumulation by *Aurantiochytrium* sp. SW1 was successfully optimized using response surface methodology (RSM). Simultaneous high yields of lipid (9.55 g/L) and DHA (44.07%) were obtained, resulting in a 17% increase in the final amount of DHA. Reproducible results were obtained when the culture was scaled-up to 5 L in a bench top bioreactor.

The isolate SW1 was previously known as *Schizochytrium* sp. SW1 based on its morphology and phylogenetic analysis. However, as described by Yokoyama & Honda (doi:10.1007/s10267-006-0362-0), the molecular phylogeny suggests that the genus *Schizochytrium* sensu lato is not a natural taxon. The first report of the polyphyly of *Schizochytrium* sensu lato by Honda et al (doi:10.1111/j.1550-7408.1999.tb05141.x) showed that members of this genus appeared in three distinct lineages. Yokoyama & Honda congregated these into three different genera: *Schizochytrium* sensu stricto, *Aurantiochytrium* and *Oblongichytrium*, based on their phenotypic characteristics as well as profiles of the PUFAs and carotenoid pigments. Based on these criteria, SW1 has been renamed as *Aurantiochytrium* SW1.

## Methods

### Microorganisms and culture conditions

*Aurantiochytrium* sp. SW1 (GenBank: KF500513) was obtained from the Microbial Physiology laboratory, School of Biosciences and Biotechnology, Universiti Kebangsaan Malaysia and maintained at room temperature on seawater nutrient agar (SNA) slant which contained 28 g/L nutrient agar (Oxoid) and 17.5 g/L sea salt. Ten single colonies of a 48 hour old culture grown on SNA were transferred into 50 mL seeding broth (in 250 mL flasks) containing 60 g/L glucose (sterilized and added separately), 2 g/L yeast extract, 8 g/L monosodium glutamate (MSG) and 6 g/L sea salt^25^. The seed culture was then incubated for 48 hours with 200 rpm agitation at 30°C. A 10% v/v inoculum was inoculated into 50 mL production medium containing sea salt, glucose, yeast extract and MSG according to the levels set in the experimental design (Table 5). The cultures were incubated for 96 hours at 30°C with 200 rpm agitation.

### Experimental design

Medium optimization was carried out using a CCD (RSM using Design Expert Version 6.0.10) with six center point replications. According to the CCD, the total number of experimental run was
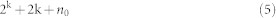
where k is the number of independent variables and *n*_0_ is the number of center point replication^28^,^29^. The following equation was employed for statistical calculations:
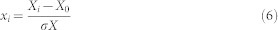
where *x_i_* is the dimensionless coded value of the variable *X_i_*, *X_0_* is the value of the *X_i_* at the center point and *δX* is the step change^30^. The behavior of the system was modeled using the following quadratic equation:
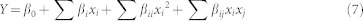
where *Y* is predicted response, *β*_0_ is the offset term, *β_i_* is the linear effect, *β_ii_* is the squared effect and *β_ij_* is the interaction effect^31^. The chosen ranges of factors are: glucose 40–80 g/L, yeast extract 0–4 g/L, MSG 0–16 g/L and sea salt 0–12 g/L. Table 5 shows the experimental design for medium optimization as well as the amount of lipid and DHA content obtained.

### Fermentation scaling-up

Validated optimal levels of each parameter were employed in a scaling-up experiment carried out in a 5 L bench-top bioreactor (Minifors-Infors HT) with a working volume of 3 L. The culture temperature was controlled at 30°C and the impeller speed was fixed at 200 rpm. The aeration was controlled at 1 vvm. Samples were collected every 12 h for biomass, lipid and DHA determination.

### Determination of dry cell weight

Cells were harvested by centrifugation at 3500 rpm (Eppendorf Centrifuge 5810R) for 15 minutes followed by two rinses with 50 ml sterile distilled water. Samples were oven-dried at 95°C to constant weight. Biomass was expressed as oven-dried weight in gram per liter of growth medium.

### Lipid extraction and fatty acid analysis

Lipid extraction was performed using chloroform-methanol (2:1, v/v), as described by Folch et al.^32^ The extract was vaporized at room temperature and dried in a vacuum desiccator until the weight was constant. Fatty acid compositions of the samples were determined as fatty acid methyl esters (FAMEs) by gas chromatography (HP 5890) equipped with a capillary column (BPX 70, 30 cm, 0.32 μm). FAME was prepared by dissolving 0.05 g of the sample in 0.95 mL hexane, and the mixture was added to 0.05 ml of 1 M sodium methoxide. The injector was maintained at 200°C. Then, 1 μl of sample was injected using helium as a carrier gas with a flow rate of 40 cm^3^min^−1^. The temperature of the GC column was gradually increased at 7°C min^−1^ from 50 (5 min hold) to 200°C (10 min hold). Fatty acids peaks were identified using Chromeleon chromatography software (Dionex, Sunnyvale, California, USA). FAMEs were identified and quantified by comparison with the retention time and peak areas of SUPELCO (Bellefonte, PA, USA).

## Author Contributions

V.M. designed and performed the experiments, interpreted the data and drafted the manuscript, M.S.K. conceived of the study, participated in its design and helped to draft the manuscript, A.A.H. conceived of the study, participated in its design and coordination, supervised all experimental procedures and helped to draft the manuscript.

## Figures and Tables

**Table 1:** ANOVA for lipid accumulation

Source	Sum of squares	Degree of freedom	Mean^2^	F value	P > F	Coefficient estimate
**Block**	26.98	2	13.49			
**Model**	**216.41**	**14**	**15.46**	**5.87**	**0.0014**	
**A**	**18.40**	**1**	**18.40**	**6.99**	**0.0202**	**0.88**
**B**	**58.35**	**1**	**58.35**	**22.17**	**0.0004**	**1.56**
**C**	11.43	1	11.43	4.34	0.0575	0.69
**D**	**34.41**	**1**	**34.41**	**13.08**	**0.0031**	**1.20**
**A^2^**	3.07	1	3.07	1.17	0.2998	−0.33
**B^2^**	40.36	1	40.36	15.34	0.0018	−1.21
**C^2^**	5.41	1	5.41	2.05	0.1754	−0.44
**D^2^**	23.42	1	23.42	8.90	0.0106	−0.92
**AB**	5.85	1	5.85	2.22	0.1597	0.60
**AC**	0.31	1	0.31	0.12	0.7383	−0.14
**AD**	6.41	1	6.41	2.44	0.1426	0.63
**BC**	0.056	1	0.056	0.021	0.8864	−0.06
**BD**	**23.95**	**1**	**23.95**	**9.10**	**0.0099**	**1.22**
**CD**	0.15	1	0.15	0.056	0.8167	−0.10
**Residuals**	34.21	13	2.63			
**Lack of fit**	**33.03**	**10**	**3.30**	**8.40**	**0.0632**	
**Pure error**	1.18	3	0.39			
**Cor total**	277.61	29				

P < 0.05 is significant.

**Table 2:** ANOVA for DHA accumulation

Source	Sum of squares	Degree of freedom	Mean^2^	F value	P > F	Coefficient estimate
**Block**	46.37	2	23.19			
**Model**	**2905.88**	**14**	**207.56**	**8.47**	**0.0002**	
**A**	37.50	1	37.50	1.53	0.2379	1.25
**B**	2.30	1	2.30	0.094	0.7643	−0.31
**C**	**153.30**	**1**	**153.30**	**6.26**	**0.0265**	**2.53**
**D**	**1053.35**	**1**	**1053.35**	**42.99**	**<0.0001**	**−6.62**
**A^2^**	0.15	1	0.15	6.258E-3	0.9381	0.08
**B^2^**	181.64	1	181.64	7.41	0.0174	2.57
**C^2^**	634.40	1	634.40	25.89	0.0002	4.81
**D^2^**	624.94	1	624.94	25.51	0.0002	4.77
**AB**	2.81	1	2.81	0.11	0.7405	−0.42
**AC**	32.23	1	32.23	1.32	0.2721	−1.42
**AD**	1.72	1	1.72	0.070	0.7951	−0.33
**BC**	0.020	1	0.020	8.178E-4	0.9776	0.04
**BD**	**359.40**	**1**	**359.40**	**14.67**	**0.0021**	**−4.74**
**CD**	60.63	1	60.63	2.47	0.1397	−1.95
**Residuals**	318.51	13	24.50			
**Lack of Fit**	**299.05**	**10**	**29.91**	**4.61**	**0.1175**	
**Pure error**	19.46	3	6.49			
**Cor total**	3270.76	29				

P < 0.05 is significant.

**Table 3:** Mineral content of artificial sea water

Element	Concentration in sea salt solution (35 g/L sea salt) (mg/L)	Concentration in culture medium (6 g/L sea salt) (mg/L)
**Be 313.107**	0.0814	0.0140
**Cd 228.802**	0.0813	0.0139
**Ca 317.933**	202.4837	34.7115
**Cr 267.716**	0.0596	0.0102
**Co 228.616**	0.0637	0.0109
**Cu 327.393**	0.2538	0.0435
**Fe 238.204**	0.0829	0.0142
**Li 670.784**	0.1990	0.0341
**Mg 285.213**	180.6768	30.9732
**Mn 257.610**	0.0646	0.0111
**Mo 202.031**	0.1618	0.0277
**Ni 231.604**	0.0633	0.0109
**Sb 206.836**	0.0519	0.0089
**Se 196.026**	0.0707	0.0121
**Tl 190.801**	0.0672	0.0115
**V 290.880**	0.0734	0.0126
**Zn 206.200**	0.1178	0.0202

**Table 4:** Summary of biomass production and DHA content of various thraustochytrids in comparison to SW1 (YE: yeast extract, MSG: monosodium glutamate)

Strain	Carbon source	Nitrogen source	% DHA/TFA	Biomass (g/L)	Reference
*Aurantiochytrium* sp. KRS101*	Glucose	YE, NH_4_C_2_H_3_O_2_	±40	±33	[27]
***Aurantiochytrium* sp. KRS101**	Glucose	YE, NH_4_C_2_H_3_O_2_	±34	24.8	[27]
***S. mangrovei* G-13**	Glucose	YE, Peptone	28	14	[33]
***Schizochytrium* sp. SR21**	Glucose	(NH_4_)_2_SO_4_	33.3–38.6	21.9–59.2	[34]
***Schizochytrium* sp. KH 105**	Glucose	Waste water from barley distillery	25.8	±26	[35]
***Aurantiochytrium* sp. SW1**	Glucose	YE, MSG	47.9	24.5	This study

*fed-batch cultivation.

**Table 5:** Experimental design for medium optimization

Run	A: Glucose	B: Yeast extract	C: MSG	D: Sea salt	Lipid (g/L)	DHA (% g/g lipid)
**1**	80.00	4.00	0.00	12.00	9.51	18.66
**2**	40.00	4.00	0.00	0.00	2.24	39.63
**3**	40.00	4.00	16.00	12.00	5.09	26.68
**4**	40.00	0.00	16.00	0.00	2.14	41.48
**5**	60.00	2.00	8.00	6.00	4.92	16.86
**6**	60.00	2.00	8.00	6.00	6.44	21.11
**7**	80.00	0.00	0.00	0.00	0.98	28.80
**8**	80.00	4.00	16.00	0.00	2.24	48.58
**9**	40.00	0.00	0.00	12.00	1.19	26.30
**10**	80.00	0.00	16.00	12.00	1.15	27.67
**11**	80.00	4.00	0.00	0.00	1.27	44.20
**12**	80.00	0.00	0.00	12.00	0.81	33.14
**13**	40.00	4.00	0.00	12.00	4.80	16.65
**14**	40.00	0.00	0.00	0.00	0.24	19.98
**15**	80.00	0.00	16.00	0.00	0.73	37.24
**16**	60.00	2.00	8.00	6.00	6.59	16.31
**17**	40.00	0.00	16.00	12.00	1.96	24.87
**18**	40.00	4.00	16.00	0.00	2.68	44.22
**19**	60.00	2.00	8.00	6.00	6.35	17.40
**20**	80.00	4.00	16.00	12.00	9.63	23.29
**21**	60.00	6.00	8.00	6.00	5.43	19.13
**22**	60.00	2.00	24.00	6.00	9.21	39.03
**23**	60.00	2.00	8.00	6.00	7.47	16.46
**24**	60.00	2.00	8.00	0.00	2.51	48.42
**25**	60.00	0.00	8.00	6.00	0.84	34.05
**26**	60.00	2.00	8.00	18.00	6.07	22.36
**27**	60.00	2.00	0.00	6.00	3.21	32.04
**28**	60.00	2.00	8.00	6.00	7.54	20.89
**29**	20.00	2.00	8.00	6.00	2.89	14.54
**30**	100.00	2.00	8.00	6.00	10.41	18.66
